# Whale sharks target dense prey patches of sergestid shrimp off Tanzania

**DOI:** 10.1093/plankt/fbv010

**Published:** 2015-03-17

**Authors:** Christoph A. Rohner, Amelia J. Armstrong, Simon J. Pierce, Clare E. M. Prebble, E. Fernando Cagua, Jesse E. M. Cochran, Michael L. Berumen, Anthony J. Richardson

**Affiliations:** 1 Marine Megafauna Foundation, Praia Do Tofo, Inhambane, Mozambique; 2 Oceans and Atmosphere Flagship, Csiro Marine and Atmospheric Research, Ecosciences Precinct, Dutton Park, Brisbane, QLD 4102, Australia; 3 Wild Me, Tofo Beach, Inhambane, Mozambique; 4 Red Sea Research Center, King Abdullah University of Science and Technology, Thuwal 23955, Saudi Arabia; 5 Centre for Applications in Natural Resource Mathematics (CARM), School of Mathematics and Physics, The University of Queensland, St Lucia, QLD 4072, Australia

**Keywords:** tropical zooplankton, elasmobranch, filter feeding, biomass threshold

## Abstract

Large planktivores require high-density prey patches to make feeding energetically viable. This is a major challenge for species living in tropical and subtropical seas, such as whale sharks *Rhincodon typus*. Here, we characterize zooplankton biomass, size structure and taxonomic composition from whale shark feeding events and background samples at Mafia Island, Tanzania. The majority of whale sharks were feeding (73%, 380 of 524 observations), with the most common behaviour being active surface feeding (87%). We used 20 samples collected from immediately adjacent to feeding sharks and an additional 202 background samples for comparison to show that plankton biomass was ∼10 times higher in patches where whale sharks were feeding (25 vs. 2.6 mg m^−3^). Taxonomic analyses of samples showed that the large sergestid *Lucifer hanseni* (∼10 mm) dominated while sharks were feeding, accounting for ∼50% of identified items, while copepods (<2 mm) dominated background samples. The size structure was skewed towards larger animals representative of *L.hanseni* in feeding samples. Thus, whale sharks at Mafia Island target patches of dense, large, zooplankton dominated by sergestids. Large planktivores, such as whale sharks, which generally inhabit warm oligotrophic waters, aggregate in areas where they can feed on dense prey to obtain sufficient energy.

## INTRODUCTION

Large marine animals (>1 t in weight) feeding on small prey (<1 g) need to consume vast amounts of food to sustain their energy demands. Most large planktivores therefore forage in areas of high prey density in high latitudes. For example, southern hemisphere humpback whales *Megaptera novaeangliae* migrate 10 000 km annually from their tropical breeding grounds to foraging areas in highly productive Antarctic waters (Zerbini *et al*., 2006). By contrast, large planktivorous fishes from the tropics and subtropics are not able to exploit these productive yet cold areas because of their ectothermic metabolism. These species face the challenge of finding prey in comparatively nutrient-poor waters (Sarmiento and Gruber, 2006; Richardson, 2008), where plankton abundance is highly variable in space and time (Lalli and Parsons, 1997). Only a handful of these large planktivorous fish species exist in the tropics and subtropics, including the whale sharks *Rhincodon typus* that reach up to 18.8 m in total length (McClain *et al*., 2015), manta rays *Manta birostris* and *Manta alfredi* (>7 and 5 m disc width, respectively; Marshall *et al*., 2009) and some *Mobula* species (Couturier *et al*., 2012). Here, we focus on the largest of all fishes, the whale shark.

Although whale sharks range through a variety of habitats in tropical and subtropical waters, from bathypelagic depths to the coastal surf zone (Brunnschweiler *et al*., 2009; Hueter *et al*., 2013), predictable feeding aggregations are known only from a small number of locations (Rowat and Brooks, 2012). These aggregations are typically dominated by juvenile sharks from ∼3 to 9 m in total length (C. A. Rohner *et al*., unpublished results). Whale sharks feed on a variety of prey species (Colman, 1997; Nelson and Eckert, 2007; Rohner *et al*., 2013a); however, many of these ephemeral aggregations of whale sharks appear to target specific prey items. Major prey items include mysids off the coast of South Africa and Mozambique (Rohner *et al*., 2013a), fish spawn off Qatar, the Yucatan coast of Mexico and Belize (Heyman *et al*., 2001; de la Parra Venegas *et al*., 2011; Robinson *et al*., 2013), copepods in the Baja California of Mexico (Clarke and Nelson, 1997; Nelson and Eckert, 2007), brachyuran eggs off Christmas Island (Meekan *et al*., 2009), chaetognaths off Djibouti (Rowat *et al*., 2011), and pseudeuphausiids at Ningaloo Reef, Australia (Jarman and Wilson, 2004).

Whale shark prey items can quickly be identified at most feeding sites; however, a detailed understanding of the zooplankton community is lacking from all of these aggregation areas. This information is needed to characterize their feeding habitat and could improve predictions of the location and timing of whale shark aggregations. Whale sharks appear to move far in search of good feeding locations and once there are presumed to forage in high-density prey patches, but the yield of such patches has only rarely been quantified. Here, we investigated the relationship between whale shark feeding ecology and the zooplankton community off Mafia Island, Tanzania. This is a typical coastal whale shark aggregation, dominated by juvenile sharks (Rohner *et al*. in revision). We measured zooplankton composition, biomass and size spectra during both whale shark feeding events and in background samples from the same area. We hypothesized that zooplankton in whale shark feeding areas would: (i) have a higher biomass, (ii) have a larger mean size of zooplankton and (iii) comprise mainly macrozooplankton, in comparison with adjacent areas in which whale sharks were not feeding at that time.

## METHOD

### Study site and sampling design

**Fig. 1.**
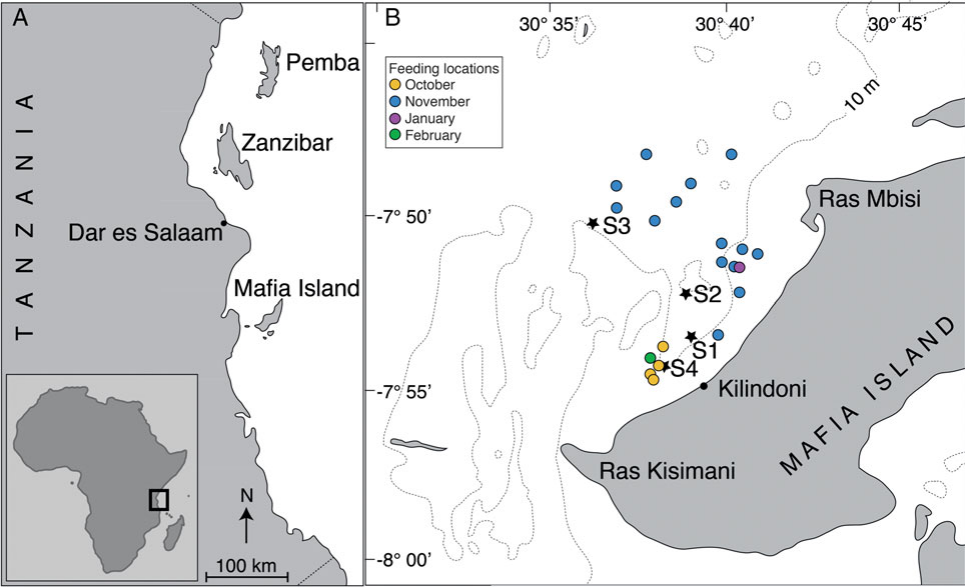
Study location with (**A**) the coastline of Tanzania showing Mafia Island and other major locations, and (**B**) a close-up of Kilindoni Bay, indicating the 10 m depth contour, regular sampling stations (S1–S4), and locations of plankton samples collected near feeding whale sharks and analysed.

The study was conducted in Kilindoni Bay, off Mafia Island, Tanzania (7.9°S, 39.6°E; Fig. 1). The bay, which does not exceed 30 m depth, extends from Ras Kisimani in the south to Ras Mbisi to the north. The intertidal zone is up to ∼1 km wide and mangroves line the bay, except for the area off Kilindoni town. The substrate in the bay is mostly sand, with a few dispersed coral reef areas and some mud and sea grass close to the coast.

Fieldwork was conducted between 17 October 2012 and 15 March 2013. Our 102 boat trips started and ended near Kilindoni town (Fig. 1) and were designed to find whale sharks in the bay and visit four stations (S1–S4), which we considered would reflect the background zooplankton community. S1 and S4 were in shallow water (∼15 m depth) close to shore, S3 was ∼6 km offshore in 30 m deep water and S2 was at medium depth and distance from shore, reflecting a spread of conditions in the bay. Mean survey duration was 238 min (±76 min SD, range = 47–540 min) and mean trip distance was 32 km (±9 km SD, range = 7–51 km).

### Whale shark observations

At least one observer was searching for whale sharks at all times during boat trips. Almost all whale sharks were spotted when their first dorsal fin, upper jaw or upper caudal lobe broke the surface, while occasionally birds diving into the water indicated their presence of whale sharks, or fishers informed us of sightings. When a whale shark was seen, a swimmer with a camera entered the water to collect data on the individual. Where possible, each whale shark was identified based on a photograph of the spot pattern posterior to the gills (Arzoumanian *et al*., 2005) and a unique encounter number was assigned in the global whale shark database (Wild Me, 2014). It was not possible to photograph every shark sighted, but we used the number of unique whale sharks identified photographically in all further analyses to avoid any potential double counting of individuals. This means that results presented here represent the minimum number of sharks observed.

Whale shark feeding behaviours were also observed and recorded. Feeding behaviour was classified into: (1) active surface feeding, characterized by fast swimming at the surface, often with the upper jaw, first dorsal and upper caudal lobe out of the water, and actively gulping in water; (2) passive subsurface feeding, characterized by slower swimming at depth with the mouth partially open; (3) vertical feeding, characterized by the shark gulping in water while stationary in a vertical position (Nelson and Eckert, 2007) and (4) gulp feeding when approaching the surface while doing short vertical oscillations in the top 10 m of the water column, similar, but over a narrower depth range, to yo–yo dives described by Gleiss *et al.* (Gleiss *et al.*, 2011). All plankton samples from feeding events in this study were taken during active surface feeding behaviours.

### Zooplankton collection and analyses

Plankton was collected using a 200 μm mesh net with 50 cm diameter, towed for 3 min at the surface ∼15 m behind the boat. Samples were immediately fixed in a 5% formaldehyde solution and kept until analyses in May 2013. We separated the plankton tows into two categories: (1) “Feeding”, whereby the net was towed within 5 m of one or more whale sharks actively feeding at the surface; and (2) “Background”, whereby plankton was collected at fixed locations (S1–S4). Background tows were linear and consistent in starting location, direction and duration. Feeding tows were fewer in numbers than background tows because they were opportunistic and they varied in location, depending on where whale sharks were feeding (Fig. 1).

Whale shark feeding locations off Mafia Island varied in space and time. However, the sharks routinely fed near the regular background stations (2.9 ± 1.8 km distance), which enabled us to make direct comparisons between feeding and background zooplankton communities. Prey patches that attracted feeding whale sharks were visually estimated to be only several 10 s of metres wide.

We collected 50, 52, 53, 47 and 20 samples from stations 1, 2, 3, 4 and whale shark feeding samples, respectively. We analysed biomass, composition and size structure of zooplankton samples. We used dry weight to calculate biomass of all samples and determined relative and absolute biomass as well as size structure in 19 samples using ZooScan (Gorsky *et al*., 2010). One additional feeding sample was excluded from ZooScan analysis because it had high densities of the filamentous cyanobacteria *Trichodesmium*, which is prohibitively difficult to separate from other organisms. GPS positions were recorded at the start and end location for plankton tows. We also determined the surface current by measuring the distance a round surface drone floated over a ∼3–5 min period. The filtered volume was calculated using the distance (ZPDist) and direction (ZPDir) of the plankton tow, the distance (CDist) and direction (CDir) of the surface current and the radius (*R*) of the net:

$$\eqalign{\hbox{\,filtered}\,\hbox{volume}& = \pi {R^2}\bigg( \hbox{ZPDist}\, + \,\hbox{CDist}\, \cr &\quad \times \,\cos \left( {\displaystyle{\pi \over {180}} \times {\rm (ZPDir} - \hbox{CDir)}} \right) \bigg).}$$

In the laboratory, the formalin solution was removed, samples rinsed in freshwater and then split into two equal parts with a Folsom plankton splitter. One half was placed in a pre-weighed glass Petri dish, dried for 24 h at 60°C and re-weighed. Zooplankton biomass was expressed as dry mass per filtered water volume in mg m^−3^. The other half of the sample was stored in formalin and later used for taxonomic composition and size spectrum determination. For comparison, values for wet mass from a whale shark feeding study by Motta *et al*. (Motta *et al*., 2010) were converted to dry mass using a factor of 0.171 for crustacean zooplankton (Young *et al*., 1996).

Using ZooScan, we also calculated the absolute and relative (per taxonomic group) biomass of 9 feeding samples and 10 background samples collected on the same day, except for the comparison sample on the 7 January 2013 feeding tow that was collected 1 day later. The corresponding background sample used for comparison with each feeding sample was collected from the closest regular sampling station. Preserved samples were fractioned with a Stempel pipette, and processed according to scanning methods outlined in Schultes and Lopes (Schultes and Lopes, 2009) using a 2400 dpi resolution ZooScan. In addition to separating particles in the scanning tray for a maximum time of 20 min per sample, we conducted a digital separation to reduce incidence of touching particles. We used the *Plankton Identifier* (Gorsky *et al*., 2010) software to classify particles into broad taxonomic groups, followed by a visual validation to ensure accurate classification of organisms, detritus (e.g. sand, fibres, debris) and scanning artefacts (bubbles, shadows).

The *Zooprocess* software (Gorsky *et al*., 2010) provided a suite of size measurements for each particle. Particles classified as detritus or scanning artefacts were excluded from further analysis. We converted the particle area measurement in pixels to square millimetres, and finally estimated spherical biovolumes (SBv). Normalized biomass size spectra (Platt and Denman, 1978) were calculated by summing the SBv of each particle into 50 normalized size bins. The log normalized biovolume for each bin is adjusted according to the fraction of the sample scanned and the total volume filtered by the net.

Particle sizes were determined with ZooScan and measurements in pixels were converted to square millimetres. Lengths of *Lucifer hanseni* were used to create a size spectrum for the species. The log-transformed SBv was used to compare particle sizes of feeding and background samples.

Owing to the small size and sparse distribution of prey patches and the fast movements of whale sharks, the net did not remain inside the targeted patch over the entire duration of each tow. It is therefore likely that we underestimated the biomass of these feeding patches.

We analysed the same 19 feeding and background samples as for biomass (above) and for their taxonomic composition. Samples were diluted and subsamples analysed under a stereo-microscope until at least 100 zooplankton specimens were counted (mean ± SD = 231.7 ± 115.5, range = 101–512) and abundance calculated as counts per m^−3^.

### Statistical analyses

A multi-dimensional scaling (MDS) analysis was performed in Primer (v. 6.1.6, Primer-E) using 21 taxonomic categories and lumping the 11 least-common taxa into “others”. Data were root transformed prior to MDS calculations, with 1000 restarts and a minimum stress of 0.01. A one-way analysis of similarity (ANOSIM) with 9999 permutations was performed to test for significance differences among groups.

We calculated the feeding threshold of biomass in a logistic regression, with feeding (1) or not feeding (0) as the binomial response. The threshold was defined as the value of the predictor where the response was 0.5. Input data were the same 19 feeding and background samples collected on the same day. Statistical analyses were conducted in the program R (R Development Core Team, 2013).

## RESULTS

### Whale shark feeding behaviour

Whale sharks were feeding during 73% of encounters (380 of 524 observations). Active surface feeding was the predominant behaviour observed, accounting for 86.6% of feeding encounters. On 9.5% of feeding events, sharks were actively drawing in water on the upward moves during shallow oscillatory dives as they approached the surface. Passive subsurface feeding was recorded on 4.5% of observations, and one shark (0.3%) fed vertically underneath a full fishing net that was being pulled up to the boat.

Whale sharks were encountered on 74 days in groups of up to 22 identified individuals with a mean of 7.1 (±5.0 SD) sharks per feeding group. On 6 days, only one shark was encountered. Feeding groups had significantly more individuals (8.6 ± 5.6 individuals, *n* = 39) than when sharks were not feeding (5.7 ± 3.9 individuals, *n* = 35; *t* = −2.55, df = 60.3, *P* = 0.01).

### Biomass

**Fig. 2.**
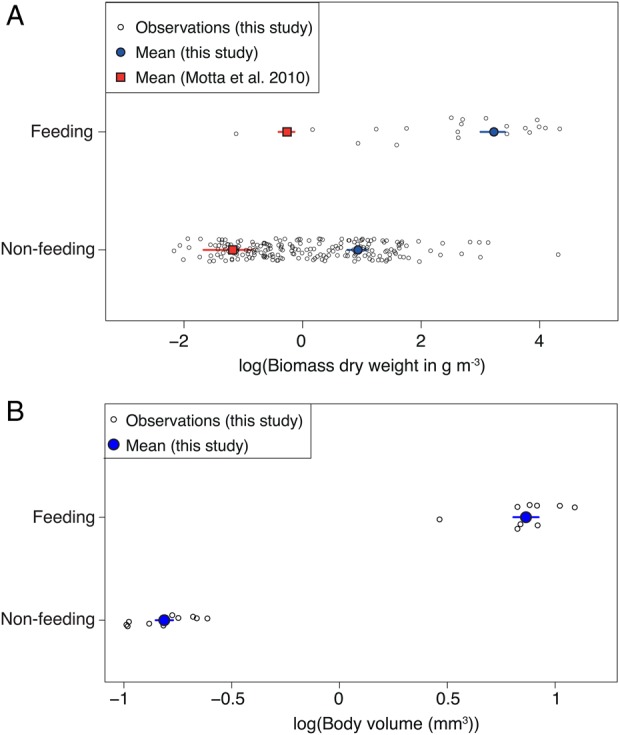
Biomass of feeding and background samples, with means ± SD, as determined by (**A**) dry mass from oven drying, including data from Motta *et al*. (Motta *et al*., 2010) and (**B**) particle size measurements from a ZooScan.

Mean zooplankton biomass calculated as dry mass from all background samples at stations 1–4 was 2.6 mg m^−3^, with the lowest mean biomass just off Kilindoni at Station 1 (1.4 ± 1.9 mg m^−3^) and the highest at Station 4 (3.7 ± 5.3 mg m^−3^). Mean zooplankton biomass in whale shark feeding samples (25.2 ± 22.8 mg m^−3^; log_10_ value = 3.23 ± 3.13) was almost an order of magnitude higher, and significantly different, than the mean biomass (2.6 mg m^−3^; log_10_ value = 0.94) from all background sampling stations (Fig. 2A; *t* = −4.43, df = 19.3, *P* < 0.001; Table I). This difference was even higher (13 times) among the directly comparable samples from the days where plankton was collected next to feeding whale sharks and also from a nearby background station (33.5 mg m^−3^ vs. 2.5 mg m^−3^; *t* = 4.2, df = 9.5, *P* = 0.002). On 16 November 2012, the highest background biomass at Station 4 was almost as high (19.7 mg m^−3^) as the mean whale shark feeding biomass; however, all whale sharks were observed further offshore where the zooplankton biomass was still three times higher. The feeding threshold was 12.4 mg m^−3^ (*r*^2^ = 0.69). Using the caloric values of whale shark feeding samples from Motta *et al*. (Motta *et al*., 2010), the energy here was 275 kJ m^−3^ of water, almost 30 times higher than in Mexico (9.4 kJ m^−3^, using the converted mean dry mass).

**Table I:** Zooplankton biomass (mg m^−3^) of background (Stations 1–4) and whale shark feeding samples as determined by oven drying

Station	Mean biomass (±SD)	*n*
1	1.4 ± 1.8	50
2	3.0 ± 10.3	52
3	2.2 ± 2.8	53
4	3.6 ± 5.3	47
All background stations	2.5 ± 6.1	202
Whale shark feeding	25.2 ± 22.8	20

### Size and community structure from ZooScan

**Fig. 3.**
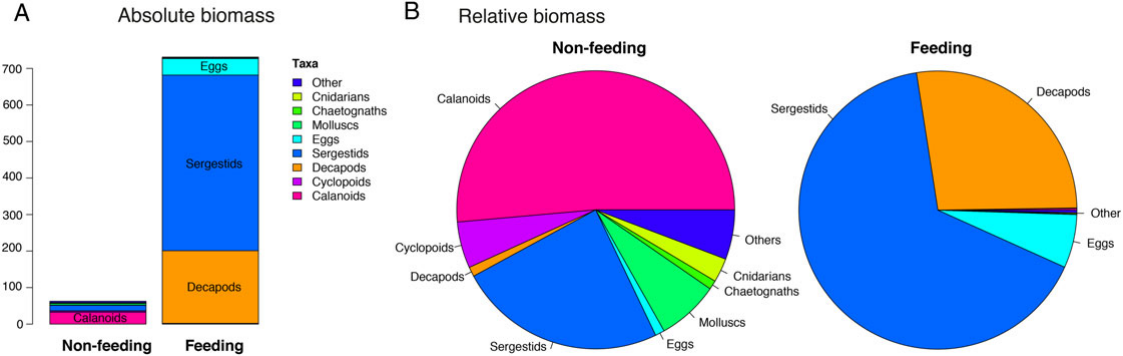
Size and taxonomic composition data determined using ZooScan for whale shark feeding- and non-feeding samples, in terms of (**A**) the absolute contributions of each taxon to biomass and (**B**) the relative contributions of taxa to biomass.

**Fig. 4.**
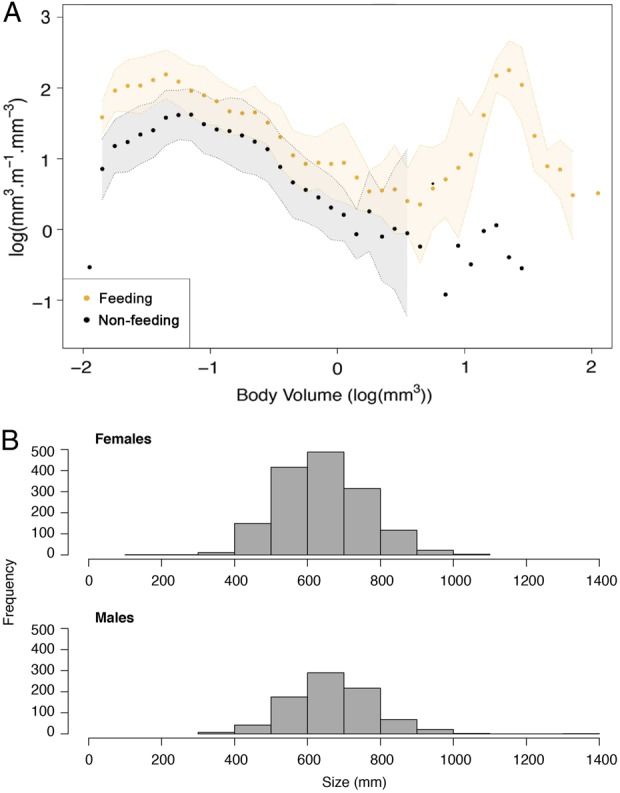
Mean size structure of (**A**) feeding and non-feeding samples with shaded 95% confidence intervals and (**B**) the frequency distribution of female and male *L.hanseni*.

The mean body volume of zooplankton from feeding samples (7.324 ± 0.879 mm^3^) was >10 times larger than that from the corresponding background samples (0.154 ± 0.015 mm^3^; *t* = 9.666, df = 18, *P* < 0.0001, Fig. 2B). Whereas copepods accounted for more than half of the biovolume (57%) in background samples, sergestids accounted for the majority of the biovolume (66%) in whale shark feeding samples (Fig. 3A and B). Large zooplankton was more abundant in feeding than in background samples (Fig. 4A). Size distributions of female and male *L.hanseni* from feeding samples were not significantly different (two-sided Kolmogorov–Smirnov test, *D* = 0.3, *P* = 0.733; Fig. 4B).

### Zooplankton composition

**Fig. 5.**
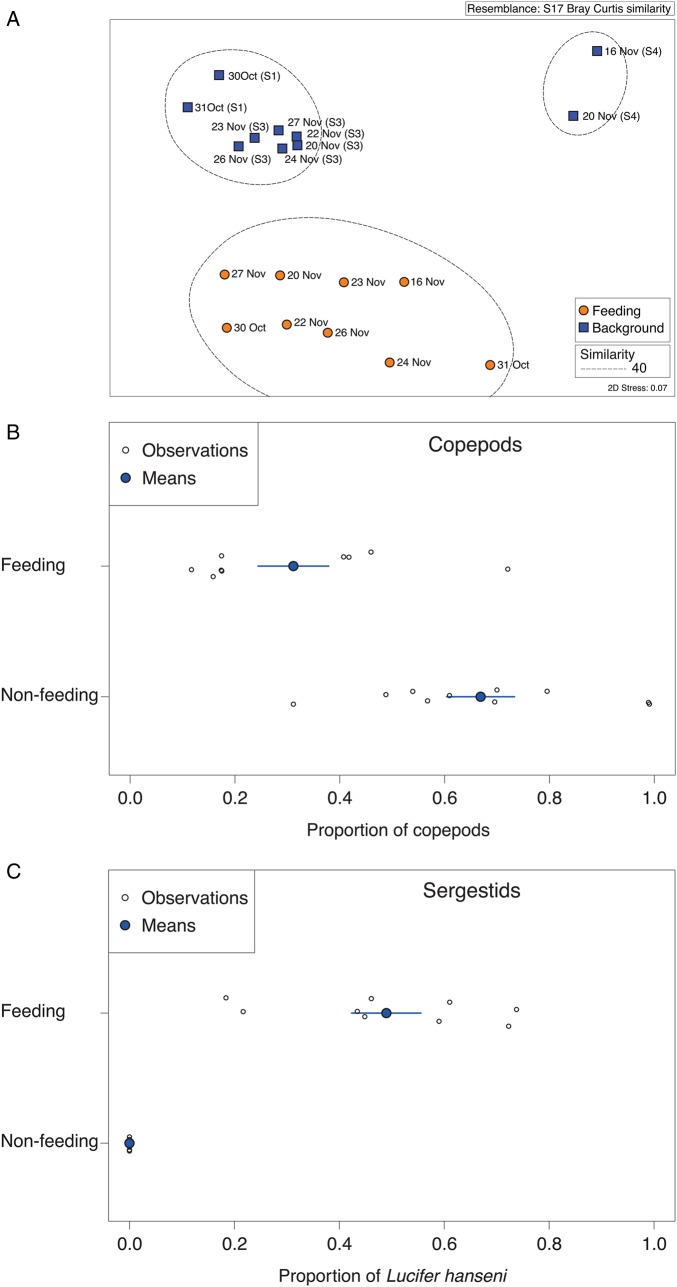
Taxonomic results determined by microscopy, with (**A**) MDS plot of taxonomic composition from feeding (orange) and background (blue) samples, with 40% similarity indicated in dashed lines; (**B**) the proportion of copepods and (**C**) the proportion of *L.hanseni*.

The taxonomic composition of samples from whale shark feeding events and from corresponding same-day background samples determined by microscopy was significantly different (Fig. 5A; ANOSIM *r* < 0.01). Background samples contained mostly copepods (67% of counted specimens; Fig. 5B). Half of all specimens identified from background samples were calanoid copepods, with 26% *Acartia* spp. and 25% juvenile calanoids (Table II). Other important copepods were the poecilostomatids *Coryaceus* spp. (9%) and *Oncea* spp*.* (3%). Of the non-copepod taxa in the background samples, eggs were the most common (17%), followed by gastropod shells (4%), brachyuran larvae (3%) and fish eggs (3%). No sergestids were found in the background samples analysed.

**Table II:** Percentages of taxonomic counts from background and whale shark feeding plankton samples, determined by microscopy counts

Taxon	Background samples	Whale shark feeding
Copepods	67.3	32.2
Calanoids	50.2	17.7
*Acartia* spp.	25.5	<1
Calanoid juvenile	24.8	16.8
Poecilostomatoids	12.3	6.8
*Corycaeus* spp.	8.8	6.3
*Oncea* spp.	3.4	<1
Cyclopoids	2.0	4.7
*Oithona* spp.	2.0	4.7
Copepod nauplii	2.6	2.8
Non-copepods	32.7	67.8
Sergestids	0	47.8
*Lucifer hanseni* f	0	29.9
*Lucifer hanseni* m	0	17.2
Oikopleurids	1.6	4.9
Eggs	17.4	3.9
Cladocerans	<1	3.0
Fish eggs	2.5	2.9
Gastropod shells	3.5	2.1
Brachyuran larvae	3.1	1.9
Decapod larvae	1.1	<1
Chaetognaths	1.0	<1

Only taxa with >1% are shown here. Also found (<1%) were harpacticoid copepods (*Microsetella* spp. and *Macrosetella* spp.), *Sapphirina* spp., fish larvae, bivalves, polychaete larvae, stomatopod larvae, *L.hanseni* mysis larvae, ostracods, calycophorans, fish scales, isopods, amphipods, mysids and scyphozoans.

**Fig. 6.**
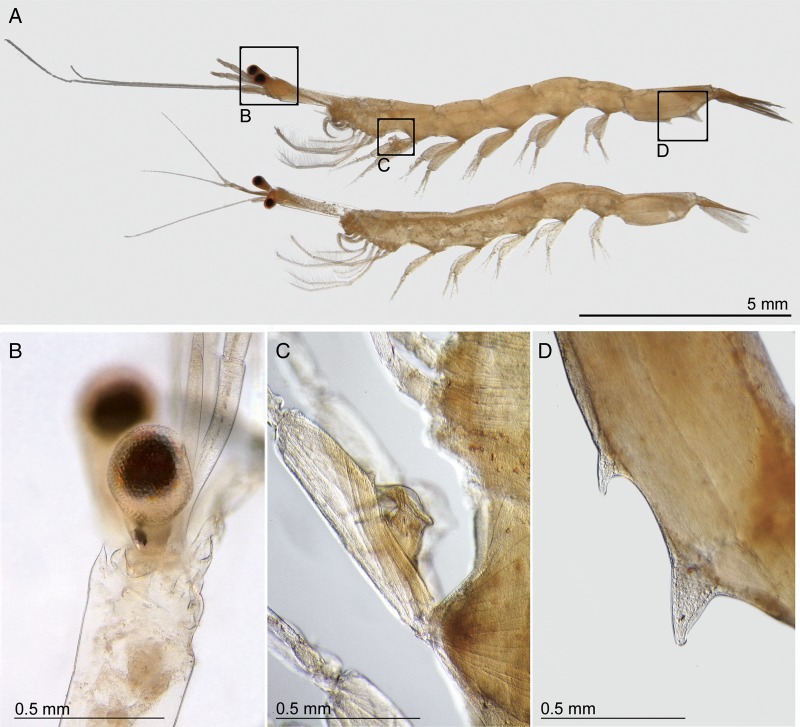
*Lucifer hanseni* from whale shark feeding tows showing (**A**) adult male (top) and female (bottom), (**B**) stalked eye with the short rostrum, (**C**) a close-up of male copulatory organ on pereiopod and (**D**) a close-up of the last abdominal segment in a male, with the two pointy processes that distinguish the males from females.

By contrast, feeding samples were dominated by non-copepod taxa (68%; Fig. 5C). Almost half of all specimens counted in whale shark feeding samples were one species of macrozooplankton, the sergestid *L.hanseni*, with 30% of the total counts being adult females and 17% adult males (Table II, Fig. 6). Other important taxa were juvenile calanoids (17%), copepods, such as *Corycaeus* spp*.* (6%), the cyclopoid *Oithona* (5%) and oiklopleurids (5%).

## DISCUSSION

Whale sharks off Mafia Island, Tanzania, target zooplankton patches characterized by a higher biomass, larger mean size and dominated by macrozooplankton compared with non-feeding areas. Whale sharks were feeding in zooplankton patches with a mean of 25 mg of dry mass per m^3^, 10 times the biomass found in background samples. Similarly, the mean particle biovolume of 7.3 mm^3^ was ∼10 times larger than in background samples. Feeding samples were dominated by the sergestid *L.hanseni*; however, we suggest that whale sharks generally do not to show prey selectivity on species, but rather target high-biomass prey patches. Whale sharks were actively surface feeding in these dense prey patches, while other feeding behaviours were rarely observed.

### Feeding behaviour

Whale sharks were mostly observed feeding actively at the surface, a behaviour previously attributed to highest density prey patches (Nelson and Eckert, 2007). Our analyses are based on such active feeding events, where zooplankton was visibly concentrated at the surface in high densities. The most common feeding behaviour in low-density prey areas was gulp feeding over the upper ∼2 m of the water column during the upward legs of shallow oscillatory dives. Subsurface passive feeding and vertical feeding were rarely seen. We could not sample zooplankton from these other behaviours and therefore the respective density thresholds could not be determined. We have successfully demonstrated, however, that prey densities were high during the most commonly observed (87%) surface feeding at Mafia Island. These dense prey patches attracted groups of feeding whale sharks that had more individuals than non-feeding groups. It is also possible that whale sharks form large aggregations below the surface, but we could not examine this as we spotted whale sharks at the surface. Surface feeding off Mafia Island is probably solely driven by food abundance, as Kilindoni Bay is shallow and thermoregulation (Thums *et al*., 2012) is unlikely to play a role here.

### Preference for high-biomass prey patches

Whale sharks off Mafia Island fed on high-density zooplankton patches. Juvenile whale sharks similarly feed in high-density prey patches in Mexico (Motta *et al*., 2010; Ketchum *et al*., 2012). We assume that whale sharks will only feed if their energetic intake exceeds the energy expended. The biomass threshold of 12.4 mg m^−3^ here was much higher than that for basking sharks (1 mg m^−3^) in the English Channel (Sims and Quayle, 1998), suggesting that whale sharks off Mafia Island only actively feed when zooplankton is highly concentrated. Compared with the converted wet mass reported in Motta *et al.* (Motta *et al.*, 2010), our difference in biomass between feeding and background samples was greater than observed off Holbox Island in Mexico (2.5 times there). The estimated energy of zooplankton per m^3^ of water was also much higher than in Mexico. This may explain why whale sharks feed for longer (∼7 h/day) in Mexico than observed here. Although whale sharks off Mafia Island were usually surface feeding (86% of feeding behaviours), it is also possible that they gain an additional important part of their dietary intake from feeding at other times or in habitats that we were unable to observe.

### Preference for macrozooplankton

A second consideration after biomass is the size of available prey. Here, we found that whale sharks off Mafia Island feed on a zooplankton community with a larger mean size than background samples. For example, on 16 November 2012, a dense patch of small copepods was found at Station 4 but no whale sharks were observed. Sharks were, however, found feeding further offshore on larger *L.hanseni*. Whale sharks do filter feed on small particles in other locations. They feed on fish eggs (∼0.8 mm in diameter) in Belize, prey smaller than the gaps in their filtering pads (Gudger, 1941; Heyman *et al*., 2001). Motta *et al*. (Motta *et al*., 2010) have proposed that whale sharks achieve this by using a cross-flow filtration technique, whereby food particles are concentrated hydrodynamically at the rear of the pharynx, rather than being strained out with the filtering pads. Thus, in at least some whale shark aggregations, prey size does not seem to matter as long as the biomass is high enough to render feeding energetically sustainable. This contrasts with our finding that whale sharks preferred large zooplankton.

The sergestid shrimp *L.hanseni* was by far the most important prey taxon in whale shark feeding samples off Mafia Island. Interestingly, these sergestids were not only the main prey of whale sharks over the 4 months of our study, but also targeted by anchovies (*Stolephorus indicus* and *Stolephorus commersonnii*) and Indian mackerel *Rastrelliger kanagurta*, which are the focus of a burgeoning local ring-net fishery (Rohner *et al*., 2013b). Whale sharks were not observed feeding on those fishes, but rather were feeding with them on *L.hanseni*. Patches of *L.hanseni*, although small in area and highly mobile, are present over several months off Mafia Island. Prey patches contained mostly adult males and females, which had a similar size distribution. Considering the low numbers of protozoea and mysis larvae in our counts, *L.hanseni* seems to aggregate in surface swarms to reproduce while the larvae develop elsewhere.

*Lucifer* shrimps are generally part of the pelagic macrozooplankton and are widely distributed in tropical and subtropical waters (Antony, 2005). The abundance, biology and ecology of *Lucifer* species in tropical waters are virtually unstudied, but there have been some laboratory studies on subtropical species. *Lucifer faxoni* has a relatively short life span of 30–40 days and reaches maturity after 29 and 19 days at 22 and 30°C, respectively (Lee *et al*., 1992). The same species undertakes daily vertical migration from close to the sediment to the surface. This can be driven by tidal fluctuations and/or by solar cycles, and the shrimp can adjust their position when moving into the water column at different tidal phases (Woodmansee, 1966). *Lucifer hanseni* also vertically migrates, and off Japan their emergence was mostly driven by tides (Oishi and Saigusa, 1997). The presence of, and potential drivers for, vertical migration of *L.hanseni* in shallow tropical bays, such as off Mafia Island, is currently unstudied. In subtropical China, *Lucifer* is most abundant in summer and has increased in abundance over the past decades, which has been attributed to warmer waters (Ma *et al*., 2009; Xu, 2010). It is thus possible that *L.hanseni* off Mafia Island is also most abundant during the warmer season when whale sharks are most frequently seen feeding at the surface.

### Do whale sharks show prey selectivity?

Although whale sharks off Mafia Island exhibited size selectivity, we believe that as a species they are likely to show only low prey selectivity. Other than avoiding or coughing up non-edible floating debris or large pieces of plastic (observations by CAR and SJP) and probably excluding small zooplankton (<∼0.5 mm in diameter, see Motta *et al*., 2010), they feed on a large variety of zooplankton prey. Whale sharks may accidentally ingest small pieces of plastic while filter feeding, but the presence and abundance of these microplastics were not investigated here. While sergestids also dominated one of the three whale shark stomach contents examined from Mozambique (Rohner *et al*., 2013a) and were the major taxon identified from some whale shark feeding events in Mexico (Motta *et al*., 2010), other important prey groups include fish spawn, chaetognaths, copepods and brachyuran larvae at other aggregation sites (see Rohner *et al*., 2013a). All of these taxa were present in our feeding samples as well, but they were likely not available in sufficient quantities for the sharks to focus on. Instead of being species selective, we suggest that whale sharks rather target high-biomass prey patches.

### Large filter feeders

Large planktivores in the tropics face the difficult challenge of finding enough food in an oligotrophic environment where their prey are sparsely distributed, and their amount and location are dynamic in time and space. Plankton-feeding whale sharks and mobulid rays aggregate in certain coastal areas *en masse* to capitalize on pulses in available prey, and likely have a varied diet over their lifetime (Couturier *et al*., 2013; Rohner *et al*., 2013a). When they find an area with a predictably high prey yield, such as in surface waters off Mafia Island during our study, whale sharks capitalize on this good feeding area. Because zooplankton prey of large, tropical elasmobranchs have only rarely been investigated, it would be interesting to gain information on prey biomass, feeding thresholds and energetic intakes at other sites and for other tropical planktivorous megafauna. Future work could also investigate their residency patterns and movements away from this site to better understand their feeding ecology and prey search strategy.

## FUNDING

Field work funding was provided by WWF Tanzania under grant CN74, the GLC Charitable Trust, and an anonymous trust. The plankton component of this work was conducted in the CSIRO Plankton Laboratory, supported by the Australian Integrated Marine Observing System (IMOS) and funded by the Australian Government through the National Collaborative Research Infrastructure Strategy and the Super Science Initiative. M.L.B. was supported by KAUST Baseline Research Funding.
